# Supplementary material for: Relationship between Microbial Composition of Sourdough and Texture, Volatile Compounds of Chinese Steamed Bread
Source: Foods. 2022 Jun 27;11(13):1908. doi: 10.3390/foods11131908 (PMC9265662; doi:10.3390/foods11131908)
Supplement: Supplementary file 1 [file foods-11-01908-s001.zip › foods-1723615-supplementary.pdf]

foods

MDPI

Supplementary Materials: The following supporting information can be downloaded at: www.mdpi.com/article/10.3390/foods11131908/s1, Figure S1: Growth ability of LAB strains isolated from Gansu sourdough; Figure S2: Changes in the pH of culture medium after growing with LAB strains; Figure S3: Comparison of the growth ability of LAB isolated from Xinjiang sourdough; Figure S4: Comparison of the acidity of LAB isolated from Xinjiang sourdough.

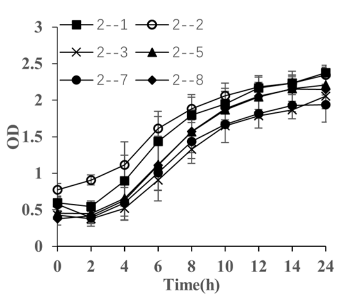

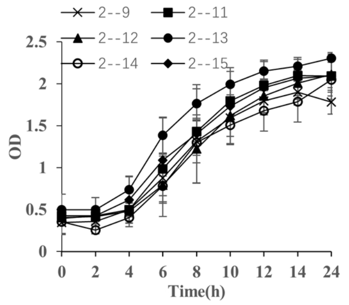

a b

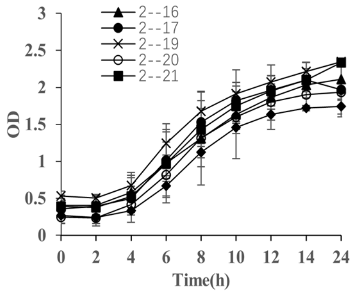

c

Figure S1. Growth ability of LAB strains isolated from Gansu sourdough. (a)The growth capacity of strain 2-1, 2-2, 2-3, 2-5, 2-7 and 2-8 was compared. (b) The growth capacity of strain2-9,2-11,2-12,2-13,2-14 and 2-15 was compared. (c) The growth capacity of strain2-16,2-17,2-19,2-20,2-21 and 2-22 was compared.

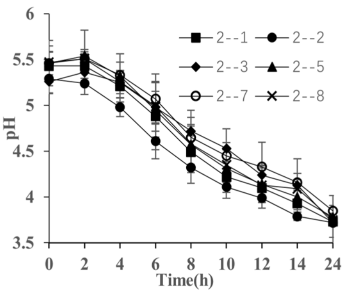

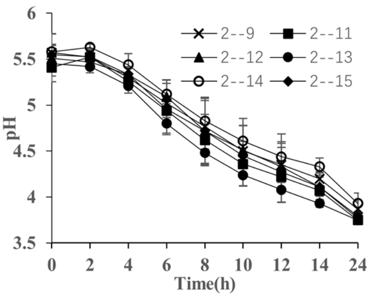

a b

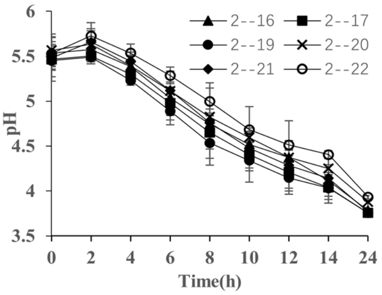

c

Figure S2. Changes in the pH of culture medium after growing with LAB strains. (a) The pH values of strain 2-1, 2-2, 2-3, 2-5, 2-7 and 2-8 were compared. (b) The pH values of strain 2-9,2-11,2-12,2-13,2-14 and 2-15 were compared. (c) The pH values of strain 2-16,2-17,2-19,2-20,2-21 and 2-22 were compared.

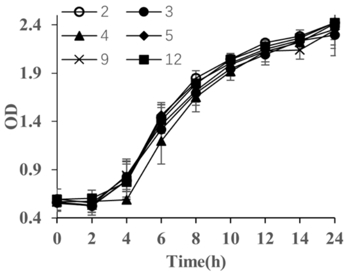

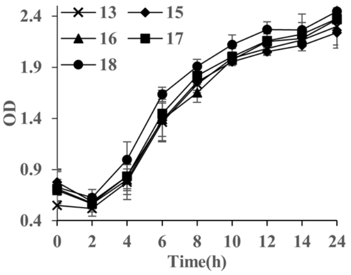

a b

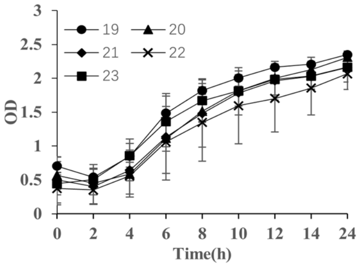

c

Figure S3. Comparison of the growth ability of LAB isolated from Xinjiang sourdough. (a) The growth capacity of strain 2, 3, 4,5, 9 and 12 was compared. (b) The growth capacity of strain13,15,16,17 and 18 was compared. (c) The growth capacity of strain 19,20,21,22 and 23 was compared.

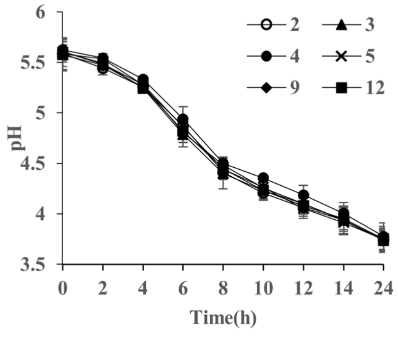

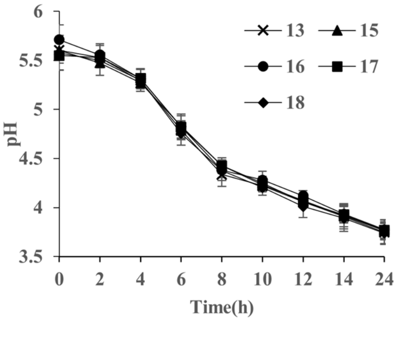

a b

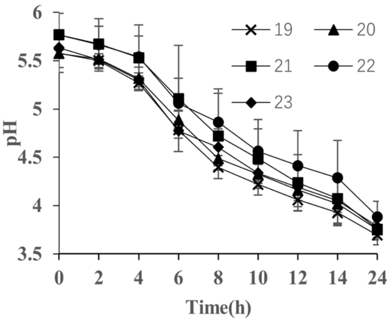

c

Figure S4. Comparison of the acidity of LAB isolated from Xinjiang sourdough. (a) The pH values of strain 2, 3, 4,5, 9 and 12 were compared. (b) The pH values of strain 13,15,16,17 and 18 were compared. (c) The pH values of strain19,20,21,22 and 23 were compared.
